# Chlorinated emodin as a natural antibacterial agent against drug-resistant bacteria through dual influence on bacterial cell membranes and DNA

**DOI:** 10.1038/s41598-017-12905-3

**Published:** 2017-10-05

**Authors:** Feixia Duan, Guang Xin, Hai Niu, Wen Huang

**Affiliations:** 1 Laboratory of Ethnopharmacology, Institute for Nanobiomedical Technology and Membrane Biology, West China Hospital, West China Medical School, Sichuan University, Chengdu, Sichuan 610041 China; 2 Department of Food Engineering, Sichuan University, Chengdu, Sichuan 610065 P.R. China; 3 College of Mathematics, Sichuan University, Chengdu, 610064 P.R. China

## Abstract

The rise in infections caused by drug-resistant pathogens and a lack of effective medicines requires the discovery of new antibacterial agents. Naturally chlorinated emodin 1,3,8-trihydroxy-4-chloro-6-methyl-anthraquinone (CE) from fungi and lichens was found to markedly inhibit the growth of Gram-positive bacteria, especially common drug-resistant bacterial strains, including methicillin-resistant *Staphylococcus aureus* (MRSA) and vancomycin-resistant *Enterococcus faecium* (VRE). CE was confirmed to cause significant potassium leakage, cell membrane depolarization and damage to the selective permeability of cell membranes in bacterial cells, resulting in bacterial cell death. In addition, CE was shown to have a strong electrostatic interaction with bacterial DNA and induce DNA condensation. Thus, CE is a promising natural antibacterial pharmacophore against Gram-positive bacteria, especially common drug-resistant MRSA and VRE isolates, with a dual antibacterial mechanism that damages bacterial cell membranes and DNA.

## Introduction

**Figure 1:**
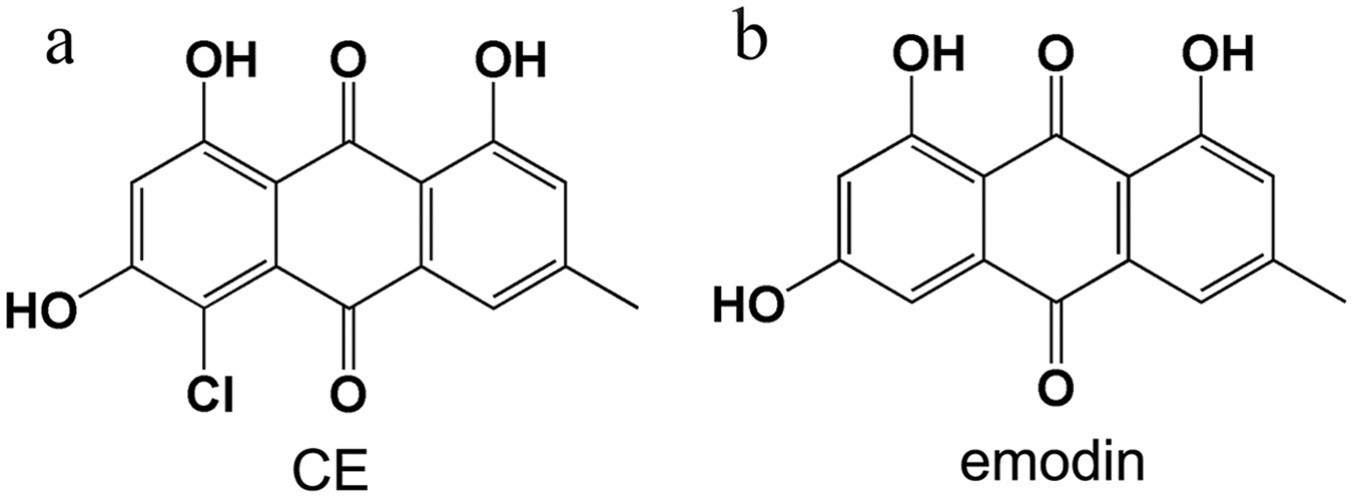
The chemical structures of CE and emodin. (**a**) CE; (**b**) emodin.

The emergence of drug-resistant bacteria and a lack of effective antibacterial agents in clinical practice has increasingly put public health at serious risk^1–6^. New antibacterial agents are urgently needed to combat this crisis, and natural bioactive compounds are important sources for new antibacterial pharmacophores. 1,3,8-Trihydroxy-4-chloro-6-methyl-anthraquinone (CE, Fig. 1a) is a bioactive compound from the lichen *Nephroma laevigatum* ^7^ and is a metabolic product of *Fusarium aquaeductuum* WC5528^8^. CE is also a naturally occurring chlorinated derivative of emodin (Fig. 1b) from the traditional Chinese herbal *Polygonum cuspidatum* roots and *Rheum palmatum* L.^9^. However, the antibacterial activity of CE remains unknown.

The bacterial cell membrane is essential for the survival and multiplication of bacteria. The membrane establishes a selectively permeable barrier to maintain cellular homeostasis^10–12^. The induction of membrane permeabilization and depolarization have been reported to be important antibacterial mechanisms^13–15^. The selection for resistance to membrane-active antibacterial agents would require a modification of the charge of membrane lipids or to the membrane constituents, which is poorly compatible with the survival of bacteria^15^. Thus, the cell membrane is a promising target for novel antibacterial agents against drug-resistant bacteria. In addition, the binding of small molecules to DNA can directly induce DNA cleavage and degradation and potentially interfere with the interaction between DNA and protein, leading to bacterial cell death^12,16,17^.

In this study, we evaluated the antibacterial activity of CE against 181 strains of laboratory and clinically isolated bacterial strains, including the common drug-resistant bacteria methicillin-resistant *Staphylococcus aureus* (MRSA), methicillin-resistant *Staphylococcus epidermidis* (MRSE) and vancomycin-resistant *Enterococcus faecalis* (VRE). We found that CE exerted potent antibacterial activity against these drug-resistant bacteria. The results revealed that CE, which affects the functions of the bacterial membrane and DNA, could be a promising natural antibacterial agent to combat drug-resistant bacteria.

## Results

### CE inhibits the growth of multiple drug-resistant Gram-positive bacteria

CE was initially found to inhibit the growth of 6 Gram-positive strains of laboratory bacteria, *S. aureus* ATCC 29213, *S. aureus* ATCC 6538, *Bacillus cereus* ATCC 10231, *Brevibacillus laterosporus* ATCC 64, *Streptococcus pneumonia* ATCC 49619 and *Enterococcus faecium* AfCC 29212 (Table 1). Impressively, the minimum inhibitory concentration (MIC) value of CE against *S. aureus* ATCC 29213 was assayed to be 4 μg/ml (Table 1), which is comparable to that of cefoxitin, a commercial antibiotic. The MIC values of emodin against *S. aureus* were assayed to be 256 μg/ml (Table 1), approximately 64-fold higher than that of CE.

**Table 1:** The MIC values of **CE** against laboratory and clinically isolated bacterial strains^a^.

Microorganism (number of isolates)	MIC (μg/ml)		
	**CE**	emodin	positive control
*S. aureus* ATCC 6538	16	256	2^f^
*S. aureus* ATCC 29213	4	256	2^g^
MSSA^b^ (15)	2–32	—	0.5–2^g^
MSSE^c^ (15)	8–64	—	1–2^g^
MRSA (15)	2–32	>256	128^g^
MRSE (15)	32–64	>256	128^g^
*E. faecalis* AfCC 29212	256	>256	2^f^
VS*-E. faecalis* ^d^ (15)	16–256	—	0.5–2^f^
VS*-E. faecium* ^*e*^ (15)	256	—	0.5^f^
VRE (15)	8–128	>256	>256^f^
*B. cereus* ATCC 10231	4	256	2^f^
*B. laterosporus* ATCC 64	4	256	2^f^
*S. pneumoniae* ATCC 49619	128	>256	0.25–1^h^
*S. pneumoniae* (18)	128	—	8–1000^h^
*Peptostreptococcus* (18)	2–256	—	0.062–256^j^
*E. coli* ATCC 25922	>256	>256	4^i^
*E. coli* PQ 37	4	—	—
*P. aeruginosa* ATCC 27853	>256	>256	4^i^
*P. aeruginosa* (12)	>256	—	2–256^i^
*B. fragilis* ATCC 25285	128	>256	0. 5–2^j^
*B. fragilis* (18)	2–128	—	0.03− > 256^j^

^a^MIC values were determined by agar dilution assay.

^b^Methicillin-sensitive *S. aureus*.

^c^Methicillin-sensitive *S. epidermidis*.

^d^Vancomycin-sensitive *E. faecalis*.

^e^Vancomycin-sensitive *E. faecium*.

^f^Vancomycin.

^g^Cefoxitin.

^h^penicillin.

^i^piperacillin.

^j^Clindamycin.

We next evaluated the antibacterial activity of CE against 141 clinically isolated Gram-positive bacterial strains, including *S. aureus*, *S. epidermidis*, *S. pneumonia*, *Enterococcus* and *Peptostreptococcus*, especially 45 drug-resistant clinical isolates of MRSA, MRSE and VRE. The result revealed that CE inhibited the growth of all 141 Gram-positive clinical isolates (Table 1). The MIC range of CE against MRSA was determined to be 2–32 μg/ml, while that of cefoxitin was 128 μg/ml (Table 1). The MIC range of CE against VRE was assayed to be 8–128 μg/ml, with vancomycin showing no inhibitory effect on VRE, even at a concentration of 256 μg/ml (Table 1). The MIC values of CE against 18 strains of clinically isolated *Peptostreptococcus* were tested in the range of 2–256 μg/ml, similar to that of clindamycin (Table 1). The results demonstrated the remarkable antibacterial activity of CE against Gram-positive bacteria, especially drug-resistant bacterial strains, including MRSA, MRSE and VRE.

### The outer membrane of Gram-negative bacteria impedes CE’s antibacterial action

The antibacterial activity of CE against Gram-negative bacteria was also evaluated. CE completely inhibited the growth of all 18 strains of clinically isolated *Bacteroides fragilis*, and its MIC values ranged from 2 to 128 μg/ml (Table 1). However, CE showed no obvious antibacterial activity against *Escherichia coli* and *Pseudomonas aeruginosa*.

The lipopolysaccharide structure in the outer membrane (OM) of *B*. *fragilis* is different from that of other common Gram-negative bacteria, such as *E. coli* and *P. aeruginosa*, which could have led to an increase in OM permeability. Therefore, we assayed the antibacterial activity of CE against a mutant of *E. coli* with defective OM permeability (*E. coli* PQ37). CE was found to completely inhibit the growth of *E. coli* PQ37 at a concentration of 4 μg/ml (Table 1), suggesting that Gram-negative bacteria are sensitive to CE when lacking the protection of the OM barrier.

**Figure 2:**
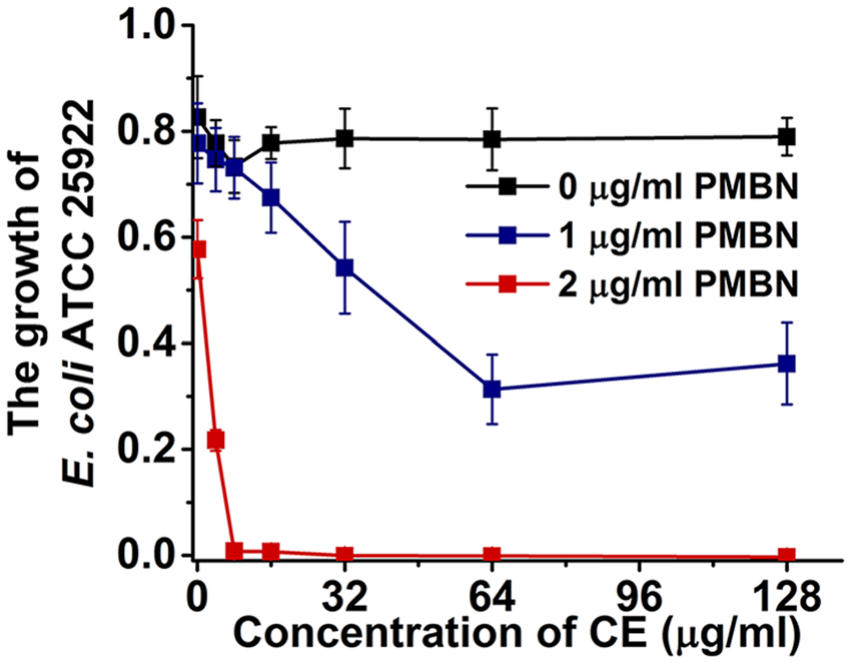
The growth of *E. coli* ATCC 25922 treated with gradual concentrations of CE in the presence of PMBN. The growth of *E. coli* ATCC 25922 was represented by the difference in the OD_i_ and OD_u_ values, where OD_i_ and OD_u_ are the optical density of inoculated medium and the corresponding uninoculated well. Plots show means of triplicates with SD.

To further assess the importance of the OM barrier on the antibacterial activity of CE, *E. coli* ATCC 25922 was treated with the permeabilizing agent Polymyxin B nonapeptide (PMBN) ^18–20^ to disrupt the OM barrier. In the presence of 2 μg/ml of PMBN, the MIC value of CE against *E. coli* ATCC 25922 was determined to be 4 μg/ml (Fig. 2), although at a concentration of 2 μg/ml of PMBN (in the absence of CE), no significant inhibitory effect on the growth of *E*. *coli* ATCC 25922 was observed (Supplementary Figure S2). The results indicated that the OM barrier contributed to the protection of Gram-negative bacteria against the antibacterial activity of CE.

### CE induces potassium leakage of bacterial cells

**Figure 3:**
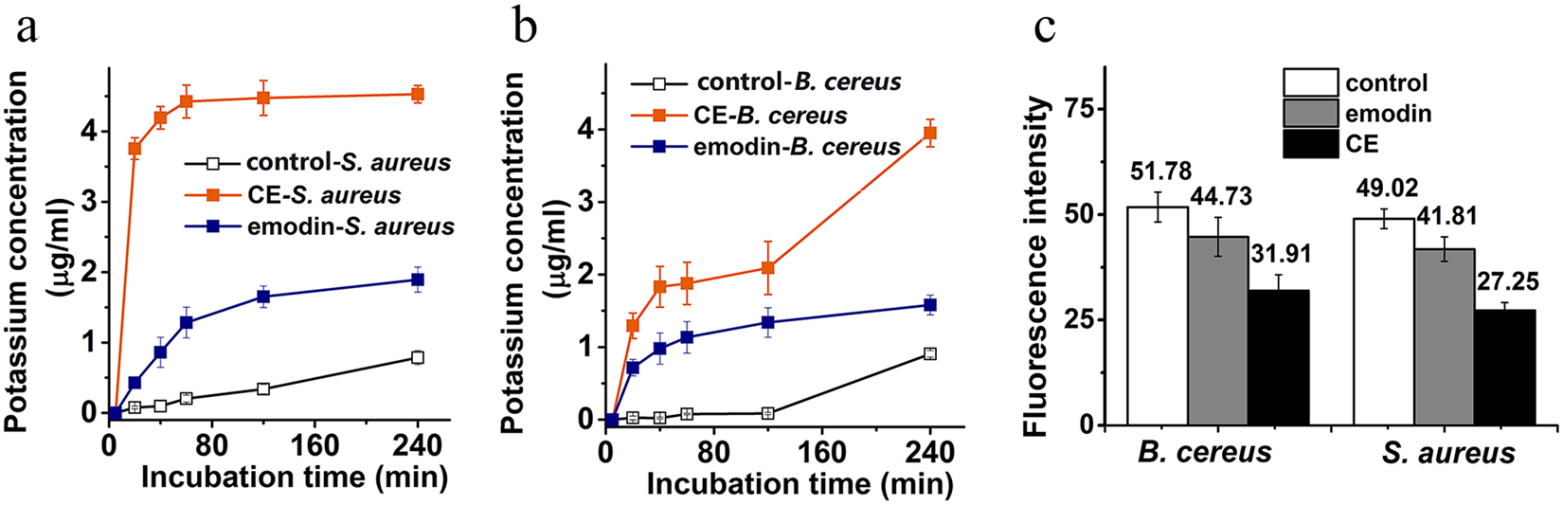
The potassium leakage and transmembrane potential of the bacterial cells treated with CE and emodin. (**a**,**b**) The K^+^ concentrations in the supernatants of *S. aureus* (**a**) and *B. cereus* (**b**) cell suspensions treated with 16 μg/ml of CE and emodin; plots show means of triplicates with SD. (**c**) T The fluorescence intensity of Rh123 stained *S. aureus* and *B. cereus* cells treated with 16 μg/ml of CE for 1 h; heights show mean values of triplicates with SD. Controls (without treatment with CE or emodin) are also shown in each panel.

The severity of potassium (K^+^) leakage in *S. aureus* and *B. cereus* cells treated with CE was determined by measuring the K^+^ concentrations in the supernatants of cell suspensions. The K^+^ concentration in the supernatants of *S. aureus* and *B. cereus* cell suspensions treated with CE increased sharply with the treatment time, while no significant increase was observed in the control group (Fig. 3a and b). The K^+^ concentration in the supernatant of the *S. aureus* cell suspension treated with 16 μg/ml CE for 1 h was 4.52 μg/ml, whereas that following treatment with 16 μg/ml emodin for 6 h was only 1.89 μg/ml (Fig. 3a). The K^+^ concentration in the supernatant of the *B. cereus* cell suspension treated with 16 μg/ml CE for 4 h was 3.65 μg/ml, and when treated with 16 μg/ml emodin for 6 h was 1.58 μg/ml (Fig. 3b). The results suggested that CE led to a significant increase in K^+^ leakage of bacterial cells, showing that CE exerted an influence on the bacterial cell membrane.

### CE causes depolarization of bacterial cell membrane

The transmembrane potential of *S. aureus* and *B. cereus* cells (5 × 10^7^ CFU/ml) was detected by staining cells with Rh123 and measuring the fluorescence intensity. The fluorescence intensity of *S. aureus* and *B. cereus* cells treated with 16 μg/ml of CE for 1 h was reduced by 44.41% and 38.37% compared with the controls (Fig. 3c), while that of bacteria cells treated with 16 μg/ml emodin decreased by only 14.71% and 13.61%, respectively. This result revealed that CE could efficiently induce the depolarization of bacterial cell membranes.

### CE increases the permeability of bacterial cell membranes

**Figure 4:**
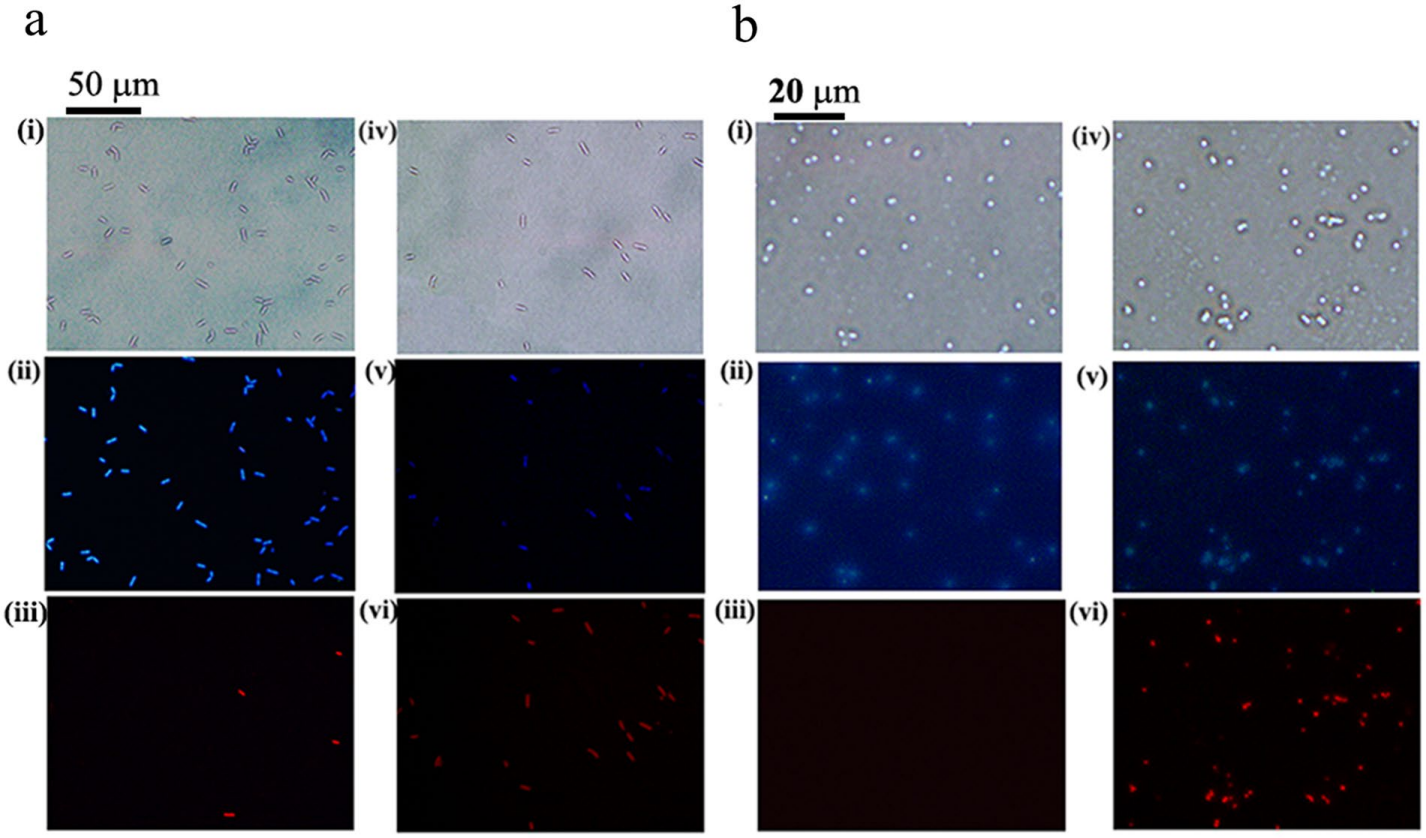
The micrograph of PI/DAPI dual-stained bacterial cells treated with CE observed with inverted fluorescence microscope. (**a**) *B. cereus* cells. (**b**) *S. aureus* cells. Samples without treatment of CE were set as control. In each panel, controls were shown in (i) (ii) and (iii), and the cells treated with 4 μg/ml of CE for 20 min in (iv) (v) and (vi); the bacterial cells observed under white light are shown in (i) and (iv); samples excited by blue light are shown in (ii) and (v), and that excited by green light in (iii) and (vi).

Dual fluorescence staining with propidium iodide (PI) and 4′,6-diamidino-2-phenylindole (DAPI) was performed to examine the selective permeability of the membranes of *S. aureus* and *B. cereus* cells (5 × 10^7^ CFU/ml) treated with CE at concentrations of 0, 4 and 16 μg/ml. In the controls, almost no red fluorescence was observed (Fig. 4a(iii), b(iii)), indicating that the membranes of normal bacterial cells prevented the entrance of PI. In both the *S. aureus* and *B. cereus* cells treated with 4 μg/ml of CE (a quarter of the MIC) for 20 min, the red fluorescence of the PI-DNA complex was clearly observed (Fig. 4a(vi), 4b(vi)) in each cell, suggesting that PI penetrated the cell membranes of CE-treated bacteria prior to cell death. The results revealed that CE could increase the permeability of bacterial cell membranes.

### CE induces static fluorescence quenching of DNA-DAPI and DNA-PI complexes

**Figure 5:**
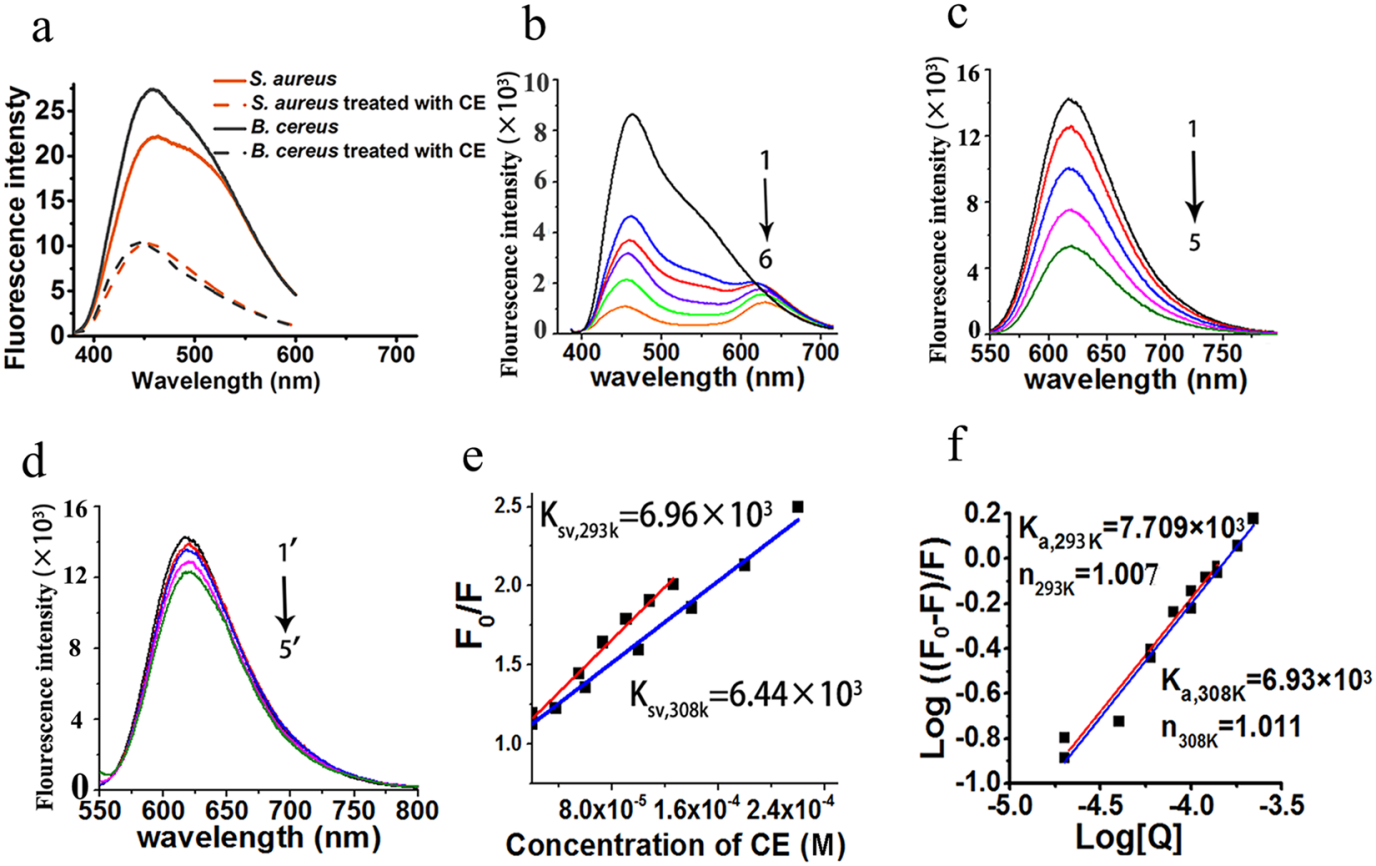
The CE-induced fluorescence quenching of DNA-PI and DNA-DAPI complexes. (**a**) The fluorescence intensity detected in the DAPI-stained *S. aureus* and *B. cereus* cells treated with 16 μg/ml of CE; the wave-length of exciting light was 358 nm; samples without treatment of CE are shown as Control. (**b**) The fluorescence spectra of DNA-DAPI complex in Tris-HCl buffer (10 mM, pH 7.2) with increasing concentrations of CE; **1**–**6** means: the concentrations of CE at 0, 10, 20, 40, 80 and 160 μM. (**c**,**d**) The fluorescence spectra of DNA-PI complex in Tris-HCl buffer (10 mM, pH 7.2) with increasing concentrations of CE (**c**) and emodin (**d**); 1–5 means: the concentrations of CE at 0, 20, 40, 60 and 80 μM; 1′-5′ means: the concentrations of emodin at 0, 20, 40, 60 and 80 μM. (**e**) The calculation of *K*_SV_ value in CE-induced fluorescence quenching of DNA-PI complex at 298 K and 308 K. (**f**) The calculation of the reaction constant (*K*_a_) and the binding site (n) of CE with the DNA-PI complex. *Micrococcus luteus* genomic DNA was used in the experiment.

During the dual fluorescence staining experiment, in both the *S. aureus* and *B. cereus* cells treated with CE, the blue fluorescence of the DAPI-DNA complex grew fainter with the increasing concentration of CE (Fig. 4a(ii), (v) and b(ii), (v), Supplementary Figure S3). The fluorescence intensity detected in both the *S. aureus* and *B. cereus* cells treated with 16 μg/ml CE declined remarkably compared with the control (Fig. 5a). These observations suggested that CE quenched the fluorescence of the DAPI-DNA complex.

To shed more light on this observed quenching effect, a fluorescence titration experiment was conducted using *Micrococcus luteus* genomic DNA in Tris-HCl buffer (10 mM, pH 7.2), where PI and DAPI were separately employed as fluorescence probes. The fluorescence spectrum detected in the solution of DNA and DAPI exhibited a major peak at 461 nm and a shoulder at approximately 543 nm due to the DAPI-DNA complex (Fig. 5b). With an increasing concentration of CE, the fluorescence intensity of the DAPI-DNA complex decreased sharply, and the major peak shifted from 461 to 452 nm, accompanied by a newly emerged peak at 630 nm (Fig. 5b). These observations highlighted the static quenching effect of CE on the fluorescence of the DNA-DAPI complex.

In the solution containing DNA and PI, the detected fluorescence spectrum was characterized by a unique peak at 617 nm due to the DNA-PI complex (Fig. 5c). With an increasing concentration of CE (20–80 μM), the fluorescence intensity of the DNA-PI complex decreased dramatically (Fig. 5c). As the concentration of CE reached 80 μM, the fluorescence intensity of the DNA-PI complex decreased by 62.7% (Fig. 5c). The quenching constants (*K*_SV_) of CE with the DNA-PI complex were estimated by the Stern-Volmer equation (S1) ^22,34^ to be 6.966 × 10^3^ at 293 K and 6.438 × 10^3^ L∙mol^−1^ at 308 K (Fig. 5e, Supplementary Figure S4), showing that the *K*_SV_ value decreased with increasing temperature. In addition, the fluorescence intensity of the DNA-PI complex slightly decreased with an increasing concentration of emodin (Fig. 5d). The results showed that CE could induce static fluorescence quenching of DNA-PI and DNA-DAPI complexes, indicating an interaction between CE and bacterial DNA.

### CE electrostatically interacts with DNA

The thermodynamic data in the static fluorescence quenching of DNA-PI complex was calculated with the Vant’t Hoff equation (S3) and the Gibbs-Helmholtz equation (S4)^21^ (Fig. 5f, Supplementary Table S1). The enthalpy change (∆H) and entropy change (∆S) were −5.335 kJ∙mol^−1^ and 55.76 J∙mol^−1^, respectively (Supplementary Table S1). The negative ∆G value indicated an exothermic interaction between CE and DNA, and the negative ∆H and positive ∆S values suggested an electrostatic interaction between CE and DNA^21–26^. This result indicated that the electrostatic effect could be the predominant driving force for the interaction of CE with DNA.

**Figure 6:**
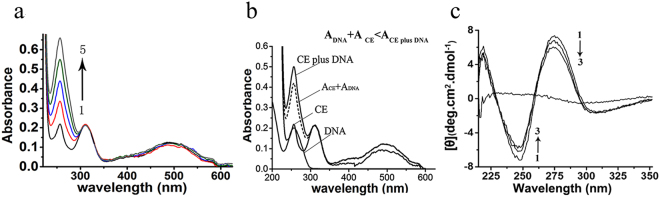
The UV-Vis spectra and CD spectra of the solution containing of CE and *Micrococcus luteus* genomic DNA. (**a**) The UV-Vis spectra of the solution containing of CE (20 μM) and increasing concentrations of DNA in Tris-HCl buffer (10 mM, pH 7.2); 1–5 means: the concentrations of DNA at 0, 5, 10, 15, 20 and 25 μM. (**b**) The sum of the individual absorbance of the CE (20 μM) and DNA (15 μM) in Tris-HCl buffer (10 mM, pH 7.2) and the individual absorbance of CE and DNA. (**c**) The CD spectra of DNA (0.01 mM) in Tris-HCl buffer (10 mM, pH 7.2)with increasing concentrations of CE; 1–3 means: the concentrations of CE at 0, 20 and 40 μM.

The interaction mode of CE with DNA was also investigated by ultraviolet-visible (UV-Vis) absorption spectroscopy. The maximum absorption of CE occurred at approximately 258 nm, and that for DNA was 260 nm (Fig. 6a). With an increasing concentration of DNA, a dramatic hyperchromic effect was observed in the spectra (Fig. 6a and b). The absorbance of the solution containing DNA (15 μM) and CE (20 μM) in Tris-HCl buffer was higher than the sum of the individual absorbance of CE and DNA (Fig. 6b). As the hyperchromic effect usually results from electrostatic binding of small molecule with DNA^22,23^, the electrostatic interaction was thus shown to be a major interaction between CE and DNA. This finding was in accordance with the results of the fluorescence quenching titration experiment.

The agarose gel electrophoresis results revealed that CE did not directly induce DNA cleavage or degradation (Supplementary Figure S6). The conformational changes in the secondary structure of *Micrococcus luteus* genomic DNA induced by CE were investigated by obtaining circular dichroism (CD) spectra. CE is optically inactive and thus has no characteristic spectrum (Fig. 6c). The CD spectrum of the *Micrococcus luteus* genomic DNA in Tris-HCl buffer consisted of a positive band at 275 nm and a negative band at 245 nm between 220 and 400 nm (Fig. 6c) due to the base stacking and helicity of B-form DNA, respectively^21,23,24^. With the increasing concentration of CE (20–40 μM), a slight drop in intensity of both the positive and negative bands was observed in the CD spectra of DNA (Fig. 6c). This phenomenon indicated that the CE-induced electrostatic attraction could alter the base packing and helical structure of DNA, leading to a transition from the extended double helical structure to a more compact conformation^25,26^. In addition, no large induced signal in the CD spectra of DNA treated with CE was observed, in agreement with the fact that CE is not an intercalator or a groove binder^27^.

### *In vitro* cytotoxicity and chromosomal aberration potential of CE

The results of the *in vitro* cytotoxicity study by an MTT test showed that CE did not inhibit the growth of Chinese hamster lung (CHL) cells at concentrations below or equal to 16 μg/ml (Supplementary Figure S7). The viability of CHL cells treated with 64 μg/ml CE for 24 h was determined to be 80.5%, whereas that of CHL cells treated with 64 μg/ml emodin was 89.8% (Supplementary Figure S7).

In the range-finding study of the *in vitro* chromosomal aberration test, CE was found to produce a visible precipitate in the culture at concentrations above 64 μg/ml. With the metabolic activation of S9, the relative population doubling (RPD) of CHL cells treated with 64 μg/ml of CE for 6 h was estimated to be approximately 85%. Without metabolic activation, the RPD of CHL cells treated with 64 μg/ml CE was 82.8% for 6 h and 77.17% for 22 h (Supplementary Figure S8). The highest concentration of CE assayed in the *in vitro* chromosomal aberration assay was determined to be 64 μg/ml according to the OECD Testing Guideline 473.

In the *in vitro* chromosomal aberration assay, compared with the reference or solvent controls, none of the tested CE concentrations (16, 32 and 64 μg/ml) produced a statistically significant increase in the total aberration frequency, and no concentration-related increase of the total aberration frequency was induced by CE (Supplementary Table S2). Cyclophosphamide (CPA), the positive control used in the test with the metabolic activation of S9, induced a remarkable increase in the incidence of aberrant metaphases (14.0% versus 2.0% in the reference controls). As expected, ethylmethanesulfonate (EMS), the positive control used in the test without metabolic activation, also caused a significant increase in the average number of aberrant metaphases. The solvent control (0.5% (vol:vol) DMSO) did not elicit a positive reaction in this test. The results suggested that in the current test conditions, CE had no genotoxic activity in CHL fibroblasts.

## Discussion

Drug-resistant bacterial infections and a lack of effective antibiotics are threats to public health. The development of novel antibacterial pharmacophores is greatly needed to fight drug-resistant infections. CE, a bioactive compound from lichen and the metabolic product of fungi^7,8^, was found to possess potent antibacterial activity against Gram-positive pathogens, including drug-resistant MRSA, MRSE and VRE isolates. The MIC values of CE against the clinically isolated MRSA strains ranged from 2 to 32 μg/ml, and those against VRE ranged from 8 to 128 μg/ml. CE is a chlorinated derivative of the traditional herbal medicine emodin. Emodin displayed almost no antibacterial activity against the drug-resistant isolates, and the MIC values of emodin against laboratory strains of *S. aureus* were approximately 64-fold higher than that of CE. Thus, CE was generally much more effective than emodin in inhibiting the growth of Gram-positive bacteria. With the exception of *B. fragilis*, CE had a decreased ability to inhibit common Gram-negative bacterial strains. Further investigation revealed that the OM barrier contributed to the lack of observed antibacterial activity of CE against Gram-negative bacteria, and the antibacterial spectrum of CE could be expanded by using permeabilizing agents such as PMBN.

K^+^ is the most abundant and essential intracellular cation in bacterial cells^28,29^. The leakage of K^+^ alters the proton motive force in the cell membrane and impairs the crucial processes situated in cell the membrane, leading to bacterial cell death^10–12,28–32^. K^+^ leakage is an important and sensitive indicator for cell membrane damage as well as membrane permeability and depolarization. In this work, we found that treating bacterial cells with CE induced a sharp leakage of K^+^ and caused cell membrane depolarization, which led to an increase in membrane permeability in both *S. aureus* and *B. cereus* cells. In addition, the influence of CE on the bacterial cell membrane was observed to be much more severe than that of emodin. These findings indicated that the ability to damage bacterial cell membranes could be a very important antibacterial mechanism of CE.

The interaction of small molecules with DNA alters the conformation and secondary structure of DNA and interferes with the accurate and precisely timed DNA-protein interactions, resulting in a cessation of bacterial growth or even cell death^16,17,23,24,32,33^. In our study, the CE-induced fluorescence quenching was initially observed in DAPI-stained *S. aureus* and *B. cereus* cells. In the fluorescence titration experiments in the solution of the *Micrococcus luteus* genomic DNA, CE was also found to induce a significant fluorescence quenching of both the DNA-DAPI and DNA-PI complexes. During the CE-induced fluorescence quenching of DNA-DAPI complex, the blue-shift of the major peak and a newly emerged peak were observed in the spectra. Besides, the *K*sv value decreased with increasing temperature in the fluorescence quenching of DNA-PI complex. These phenomena suggested that CE induced static fluorescence quenching of both the DNA-DAPI and DNA-PI complexes. In addition, the fluorescence quenching effect of emodin on the DNA-PI complex appeared to be much weaker than CE. These results confirmed the occurrence of an interaction between CE with DNA and that CE has a stronger affinity with bacterial DNA than emodin.

Intercalation, groove binding and electrostatic attraction are the three main interaction modes of small molecules with DNA^25–30^. In the CE-induced fluorescence quenching of DNA-PI complex, the thermodynamic data ∆H and ∆S were estimated to be negative (−5.335 kJ∙mol^−1^) and positive (55.76 J∙mol^−1^), respectively, indicating that electrostatic interaction could be the primary force in the binding of CE with DNA. The electrostatic interaction of CE with DNA was also verified by the hyperchromic effect observed in the UV spectra of DNA in Tris-HCl buffer incubated with CE. In addition, the absence of large induced signals in the CD spectra of DNA with an increasing concentration of CE supported the idea that CE is not an intercalator or a groove binder, and further proved the electrostatic interaction of CE with DNA. The CE-induced slight decrease in the negative (245 nm) and positive (275 nm) band intensity in the CD spectra of DNA indicated that the electrostatic interaction of CE with DNA could result in DNA condensation, which impairs essential DNA-protein interactions during the complex processes of DNA replication and transcription. In addition, the results of the agarose gel electrophoresis assay showed that the interaction of CE with DNA does not directly induce DNA cleavage or degradation.

CE exhibited similar *in vitro* toxicity as emodin on the assayed mammalian cells (CHL). At concentrations below or equal to 64 μg/ml, CE produced a negative reaction in the *in vitro* mammalian chromosomal aberration test, indicating that CE had no genotoxicity potential under the tested conditions in this study.

In conclusion, the present work indicated that CE, as a bioactive compound present in lichen and a fungus metabolic product, possesses remarkable antibacterial activity against Gram-positive bacteria, including the common drug-resistant pathogens MRSA, MRSE and VRE. CE was observed to cause potassium leakage and cell membrane depolarization of bacterial cells, and impairs the selective permeability of the bacterial cell membrane. CE also induced bacterial chromatin pycnosis by electrostatic attraction with DNA. Thus, CE is a promising natural antibacterial agent that could effectively combat drug-resistant bacteria by damaging both bacterial cell membranes and DNA.

## Methods

### Synthesis and identification of CE

The experimental procedure and the NMR spectra data of CE are shown in Supplementary Information. The chromatographic conditions and the specific purity values of CE are shown in Supplementary Fig. S1.

### Antibacterial activity assay

*S. aureus* ATCC 6538*, S. aureus* ATCC 29213, *B. laterosporus* ATCC 64*, B. cereus* ATCC 10231*, E. coli* ATCC 25922, *E. faecalis* AfCC 29212, *S. pneumoniae* ATCC 49619, *P. aeruginosa* ATCC 27853, and *B. fragilis* ATCC 25285 were purchased from the China Center of Industrial Culture Collection (CICC). *E. coli* strain PQ 37 [F- thr leu his-4 pyrD thi galE galK or galT lac∆U169 srl300::Tn10 rpoB rpsL uvrA rfa trp::Muc + sfiA::Mud(Ap, lac)ts] was obtained from the College of Food Science and Nutritional Engineering, China Agricultural University. All of the clinical isolates were obtained from the Institute of Clinical Pharmacology, Peking University. Emodin and CE were dissolved in DMSO and stored at −20 °C. Vancomycin hydrochloride, cefoxitin sodium salt, penicillin G sodium salt, piperacillin sodium salt, clindamycin 2-phosphate and ampicillin were dissolved in sterile distilled water. *E. coli* PQ 37 was cultured in LA medium. MIC values were determined using the CLSI agar dilution method and the broth micro-dilution assay for aerobic and anaerobic bacteria^35–37^. In the micro-dilution assay, the bacterial growth was calculated as the difference in the OD_i_ and OD_u_ values, where OD_i_ and OD_u_ are the optical density of inoculated medium and the corresponding uninoculated well. The MIC values were recorded as the lowest concentrations of compounds showing no bacterial growth. The MIC values were determined at least twice on separate days, with the higher value used to represent the MIC value.

### Potassium efflux measurement

The potassium efflux was measured with an atomic absorption spectrophotometer (Spectra AA 220, VARIAN, USA) according to a previously reported method^28,29^ with some modification. *S. aureus* ATCC 6538 and *B. cereus* ATCC 10231 were cultured in LB overnight at 37 °C. The cells were collected by centrifugation, then were washed and resuspended to 1 × 10^8^ CFU/ml in sodium phosphate buffer (0.1 M, pH 7.2) to regulate the cellular osmotic pressure with sodium chloride. Next, 20 ml of these suspensions was incubated with 16 μg/ml CE or 16 μg/ml emodin, and 2 ml of the cultures was withdrawn at 20, 40, 60, 120 and 240 min to examine the K^+^ concentration of the supernatant after centrifugation (2000 rpm, 10 min). As a control, 0.5% DMSO was added to a sample.

### Detection of changes in bacterial transmembrane electrical potential

Rh123 was diluted in PBS (pH 7.2) to a final concentration of 10 μg/ml and was stored in the dark at 4 °C before use. Overnight cultures of *S. aureus* ATCC 6538 and *B. cereus* ATCC 10231 were centrifuged (2000 rpm, 10 min) and pellets were resuspended to 5 × 10^7^ CFU/ml. CE and emodin were added to 10 ml of the bacterial suspensions to obtain final concentrations of 16 μg/ml before being incubated at 35 °C. As a control, 20 μl of DMSO was added to a sample. Next, 1 ml of the bacterial suspension for each sample was withdrawn after 20 min, and 10 μl of Rh123 was added to each sample. The samples were incubated at 37 °C for 30 min before being scanned with a Hitachi FL spectrophotometer F-7000 (excitation at 507 nm). The fluorescence intensities were measured at 529 nm and recorded.

### Dual fluorescence staining and PI uptake experiment

We stained the bacterial cells with DAPI and PI using previously reported methods^12,31,38,39^ with some modification. PI and DAPI were diluted in 0.1 M PBS (pH 7.2, using 0.85% NaCl to equilibrate the osmotic pressure) to final concentration of 1 mg/ml and were stored in the dark at 4 °C before use. Overnight cultures of *S. aureus* ATCC 6538 and *B. cereus* ATCC 10231 were centrifuged (2000 rpm, 10 min), and the pellets were resuspended (5 × 10^8^ cells/ml) in fresh, sterile LB medium. Next, 10 ml of bacterial cell suspension were incubated with 1.25, 2.5 or 5 μl CE (64 mg/ml in DMSO) at 35 °C. As a solvent control, 5 μl of DMSO was added into 10 ml of the bacterial suspensions. After a 30-min incubation, 50 μl of DAPI and 6.25 μl of PI were added to a 1 ml culture, and the culture was incubated at 37 °C for 10 min. Next, 1 μl cultures were aliquoted onto glass microscope slides and covered with microscope cover glass before being observed with an inverted fluorescence microscope (Olympus IX71). 1 ml aliquot of each culture was and centrifuged (2000 rpm, 10 min), and the pelleted cells were collected and resuspended in 600 μl of 20 μg/ml DAPI and incubated at 37 °C for 10 min before fluorescence intensity measurements (Hitachi FL spectrophotometer F-7000; exciting light 358 nm, emitted light 460 nm).

### Fluorescence titration experiment

*Micrococcus luteus* genomic DNA was diluted in 10 mM Tris-HCl buffer (pH 7.2). PI and DAPI were diluted in PBS (pH 7.2) to final concentrations of 1 mg/ml and 5 mg/ml, respectively, and then stored in the dark at 4 °C before use. CE was diluted in DMSO to a concentration of 0.2 mM before use. As a solvent control, 20 μl of DMSO was added to a sample. Graduated concentrations of PI or DAPI were added into the DNA Tris-HCl dilutions until the fluorescence identities of the system no longer increased. Next, increasing amounts of CE and emodin were added to the DNA Tris-HCl dilutions. All the samples were scanned with a Hitachi FL spectrophotometer F-7000. The value of *K*_SV_, apparent binding constant (*K*_*a*_), binding stoichiometry (n) and the thermodynamic data of CE in the fluorescence quenching of DNA-PI complex were estimated by Stern-Volmer equation (S1, S2), the Vant’t Hoff equation (S3) and Gibbs-Helmholtz equation (S4)^21–24^. More details are shown in Supplementary Information.

### UV-Vis measurements

*Micrococcus luteus* genomic DNA was diluted in 10 mM Tris-HCl buffer (pH 7.2). PI and DAPI were diluted in PBS (pH 7.2) to final concentrations of 22 and 30 μM, respectively, and were stored in dark at 4 °C before use. CE and emodin were diluted in DMSO to a concentration of 0.2 M before use. An increasing amount of DNA was added to the emodin, CE, PI and DAPI dilutions, and the UV-Vis absorption spectra of all the samples were measured at a wavelength range of 200–800 nm at room temperature using a DU 800 Spectrophotometer. The sum of the individual absorbances of DNA and CE were calculated^22,34^.

### Circular dichroism studies

The CD spectra of 200 μM DNA incubated with CE at concentrations of 25, 50 and 100 μM (molar ratios ([DNA]/[CE]) values of 8, 4 and 2) were measured at wavelengths between 210 and 400 nm. The optical chamber of the CD spectrometer was deoxygenated with dry nitrogen before use and maintained in a nitrogen atmosphere during experiments. CD measurements were carried out in Tris-HCl buffer (10 mM, pH 7.2) at room temperature using an AVIV 400 circular dichroism spectrometer. The changes in the CD spectra were monitored against a blank. The results were taken as ellipticity in mdeg. Scans were accumulated and automatically averaged.

### *In vitro* cytotoxicity measurements and chromosomal aberration assay

The viability of the Chinese hamster lung (CHL) fibroblast cells with and without the CE treatment was evaluated by an MTT assay. The *in vitro* chromosomal aberration assay was performed in accordance with the OECD Testing Guideline 473 (2014)^40^. The fibroblast cell line CHL/IU (ATCC CRL-1935) was from the Sichuan centre for disease control and prevention, P.R. China. CPA and EMS were used as positive controls for the *in vitro* chromosomal aberration assay with and without exogenous metabolic activation, respectively. The metabolic activation system consisted of an S9 fraction and cofactor-I. More details are shown in Supplementary Information.

All data generated or analysed during this study are included in this published article (and its Supplementary Information files).

## Electronic supplementary material

Supplementary information
