# Supplementary material for: Chlorinated emodin as a natural antibacterial agent against drug-resistant bacteria through dual influence on bacterial cell membranes and DNA
Source: Sci Rep. 2017 Oct 5;7:12721. doi: 10.1038/s41598-017-12905-3 (PMC5629251; doi:10.1038/s41598-017-12905-3)
Supplement: Supplementary file 1 — Supplementary information [file 41598_2017_12905_MOESM1_ESM.pdf]

Supplementary Information

Chlorinated emodin as natural antibacterial agent against drug-resistant bacteria through dual influence on bacterial cell membrane and DNA

Feixia Duan[a,b], Guang Xin[a], Hai Niu[a,c], Wen Huang*[a]

[a] Laboratory of Ethnopharmacology, Institute for Nanobiomedical Technology and Membrane Biology, West China Hospital, West China Medical School, Sichuan University, Chengdu, Sichuan 610041, China.

E-mail addresses: huangwen@scu.edu.cn (W. Huang).

[b] Department of Food Engineering, Sichuan University, Chengdu, Sichuan 610065, PR China.

E-mail addresses: duanfeixia@126.com (F. Duan).

[c] College of Mathematics, Sichuan University, Chengdu 610064, P.R. China.

E-mail addresses: niuhai@scu.edu.cn (H. Niu).

NMR spectra data of CE	S1
HPLC spectra of CE	S1
Supplementary Figure S1	S1
Supplementary Figure S2	S2
Supplementary Figure S3	S2
Supplementary Figure S4	S3
Supplementary Figure S5	S3
Supplementary Figure S6	S4
Supplementary Figure S7	S4
Supplementary Figure S8	S4
Supplementary Table S1	S4
Supplementary Table S2	S5
Supplementary information for methods	S6

NMR spectra data of CE

^{1}H NMR (400 MHz, DMSO) δ 12.69 (s, 1H), 11.69 (s, 1H), 7.37 (s, 1H), 7.07 (s, 1H), 6.72 (s, 1H), 2.39 (s, 3H). ^{13}C NMR (101 MHz, DMSO) δ 189.08 (s), 180.53 (s), 162.84 (s), 162.10 (s), 160.74 (s), 148.45 (s), 133.71 (s), 129.97 (s), 123.23 (s), 120.41 (s), 116.01 (s), 112.68 (s), 109.74 (s), 107.82 (s).21.585 (s). HRMS (ESI−) Calc. for $C_{15}H_8Cl_1O_5$: 303.0139 $[M-H]^-$; Found 303.0061 $[M-H]^-$.

HPLC spectra of CE

System: himadzu HPLC system (Kyoto, Japan) consisting of an LC-20AD binary pump, an SPD-20A ultraviolet detector, a SIL-20AC autosampler, a CTO-20A column oven, and a LC Solution control software

Mobile phase: 67% CAN and 33% aqueous solution of phosphoric acid (0.1% Phosphoric acid, wt:vol)

Detector: DAD at 452 nm

Column: Shimadzu Shim-pack Vp-ODS column (250 × 4.6 mm; 5 μm)

Flow rate: 1mL/min

Retention time (min): 5.304

Purity: 99.214%

The chromatographic conditions and the specific values of the purity of CE are shown in Supplementary Figure S1.

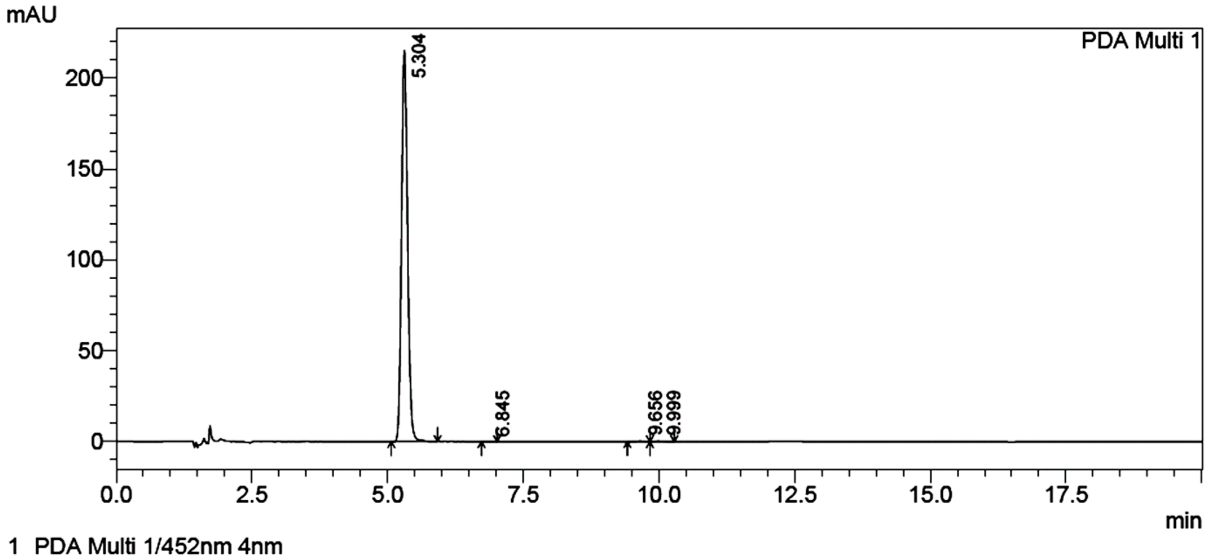

Supplementary Figure S1 HPLC analysis of CE.

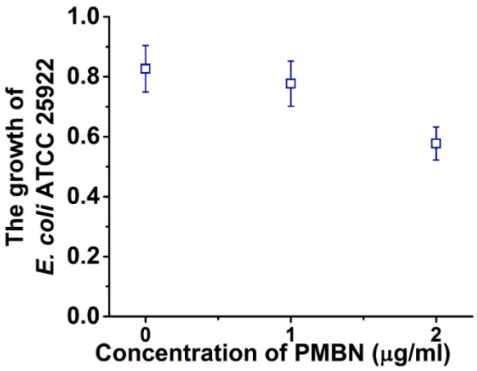

Supplementary Figure S2 The growth of *E. coli* ATCC 25922 treated with PMBN. The growth of *E. coli* ATCC 25922 were represented by the difference in the OD_i and OD_u values, where OD_i and OD_u are the optical density of inoculated medium and the corresponding uninoculated well. Plots show means of triplicates with SD.

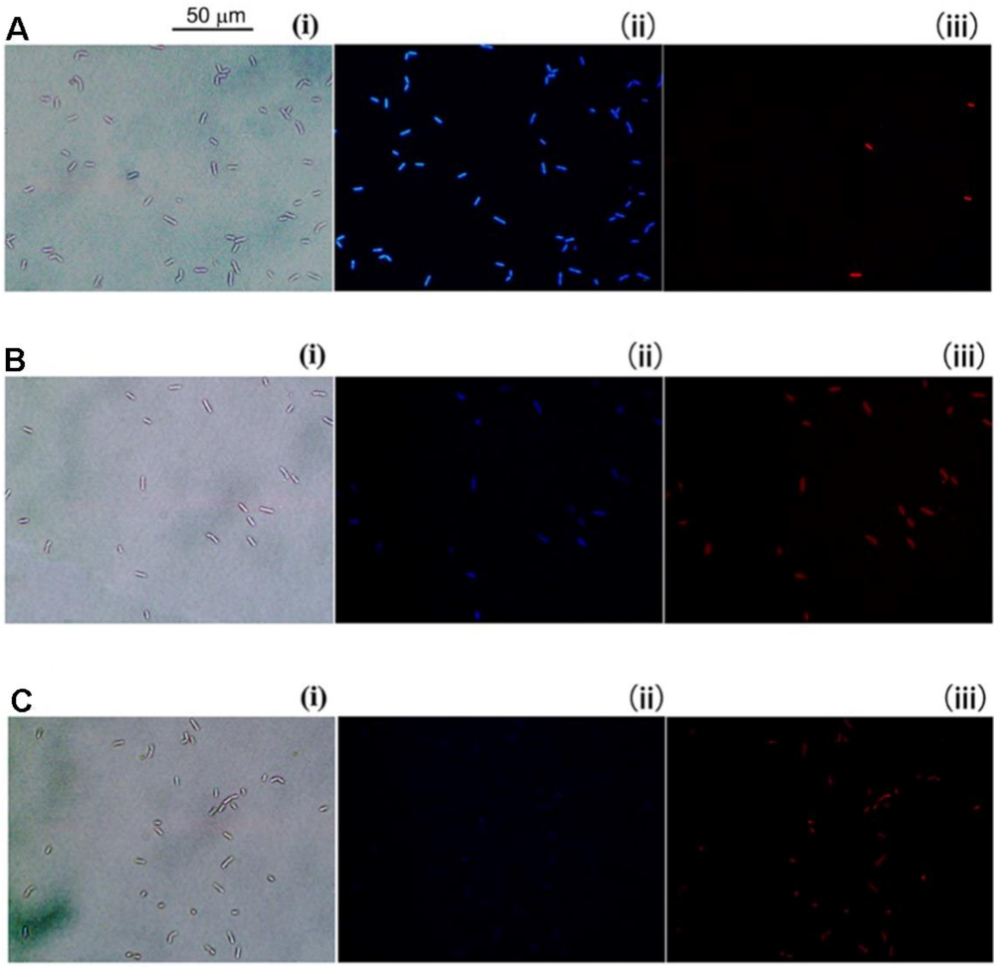

Supplementary Figure S3 The micrograph of DAPI/PI dual-stained *B. cereus* cells treated with graduate concentrations of CE. (**A**) control. *B. cereus* cells without treatment of CE were set as control. (**B-C**) *B. cereus* cells treated with 4 (**B**) and 16 µg/ml (**C**) of CE for 20 min. In each panel, samples observed under white light are shown in (i); cells excited by blue light and green light are shown in (ii) and (iii).

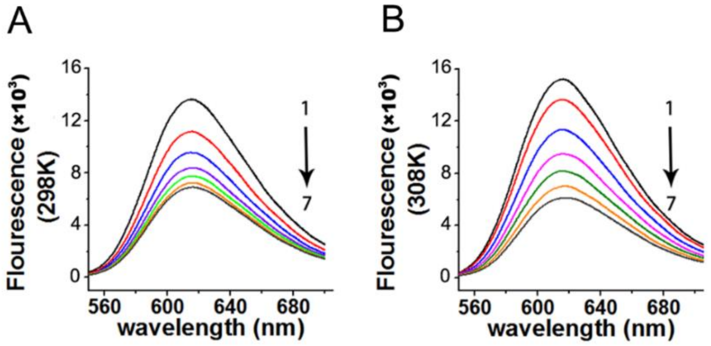

Supplementary Figure S4 The CE-induced fluorescence quenching of DNA-PI complexes at different temperature. (**A**) The fluorescence spectra of DNA-PI complex in Tris-HCl (10 mM, pH 7.2) with increasing concentrations of CE at 298 K; 1-7 means: the concentrations of CE at 0, 20, 40, 60, 80, 100 and 120 μM. (**A**) The fluorescence spectra of DNA-PI complex in Tris-HCl (10 mM, pH 7.2) with increasing concentrations of CE at 308 K; 1-7 means: the concentrations of CE at 0, 20, 60, 100, 140, 180 and 220 μM.

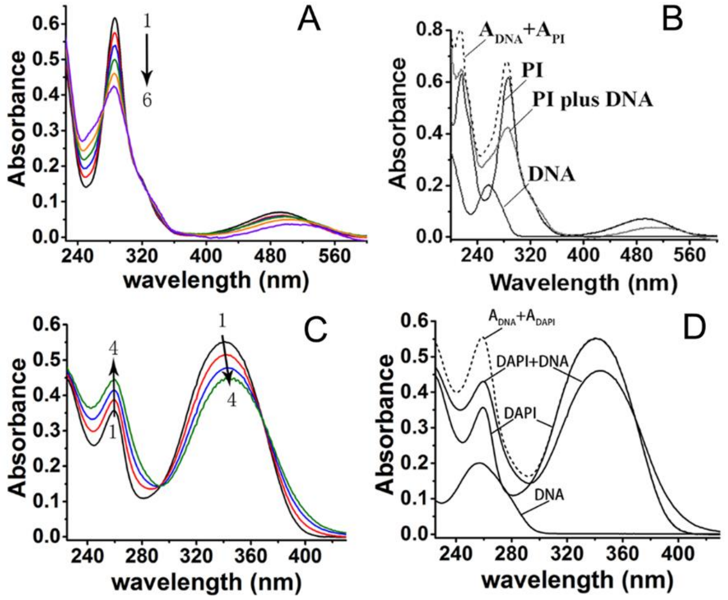

Supplementary Figure S5 The UV-Vis spectra of DNA, PI and DAPI in Tris-HCl buffer. (**A**) The UV-Vis spectra of PI (0.02 mM) in Tris-HCl buffer (10 mM, pH 7.2) with increasing concentrations of DNA; 1-6 means: the concentrations of DNA at 0, 5, 10, 15, 20 and 25 μM. (**B**) The sum of the individual absorbance of the DNA (15 μM) and PI (20 μM) in Tris-HCl buffer (10 mM, pH 7.2) and the absorbance of the solution containing DNA and PI. (**C**) The UV-Vis spectra of DAPI (30 μM) in Tris-HCl buffer (10 mM, pH 7.2) with increasing concentrations of DNA; 1-4 means: the concentrations of DNA at 0, 5, 10 and 15 μM. (**D**) The sum of the individual absorbance of the DNA (15 μM) and DAPI (30 μM) in Tris-HCl buffer (10 mM, pH 7.2) and the absorbance of the solution containing DNA and DAPI.

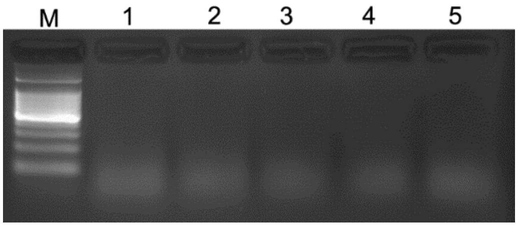

Supplementary Figure S6 The agarose gel electrophoresis of DNA treated with CE. The Salmon sperm DNA was diluted in Tris-HCl buffer (10 mM, pH 7.2) at a concentration of 100 ng/ml with increasing concentrations of CE. Lane 1-5: the concentrations of CE was 0, 16, 32, 64, 128 and 256 μg/ml. M: the DNA marker.

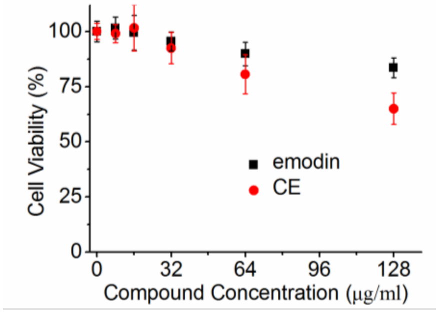

Supplementary Figure S7 The cell viability of the CHL cells treated with CE and emodin for 24 h.

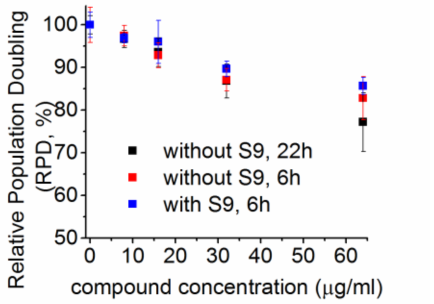

Supplementary Figure S8 The RPD of CHL cells treated with CE in the presence and absence of S9 mix.

Supplementary Table S1 The quenching constants (K_{SV}), binding constants (K_a) and the relative thermodynamic parameters in CE-induced fluorescence quenching of DNA-PI complex

Temperature (K)	K_{sv} ($\times10^3$ L·mol^{-1})	R^a	K_a ($\times10^3$ L·mol^{-1})	R^b	N	ΔH (kJ·mol^{-1})	ΔS (J·mol^{-1})	ΔG (kJ·mol^{-1})
293	6.966±0.00	0.9676	7.709±0.003	0.9722	1.007	-5.335	55.76	-21.671
308	6.438±0.00	0.9709	6.929±0.001	0.9985	1.011			-22.645

[a] R is the correlation coefficient for the K_{SV} values.

[b] R is the correlation coefficient for the K_a values.

Supplementary Table S2 The genotoxic effect of CE assessed by *in vitro* mammalian chromosomal aberration test in CHL cells

S9 mix	Concentration (μg/ml)	Treatment period (h)	CTG[a]	CTB[a]	CTE[a]	CSG[a]	CSB[a]	CSE[a]	PP[a]	ER[a]	RPD (%)	MI (%)	total aberration frequency[b] (%)
+	Reference control[c]	6 (treatment)	0/1	0/0	0/1	0/0	1/1	0/0	0/0	0/0	100.00	95.50	2.0
	Solvent control	18 (recovery)	1/0	2/5	0/0	0/0	0/0	0/0	0/0	0/0	99.17	96.67	3.5
	64		1/0	2/0	0/1	1/0	2/0	0/0	0/1	0/0	84.98	97.50	2.5
	32		0/1	2/5	0/0	0/1	0/0	0/0	0/1	0/0	90.32	96.67	3.5
	16		0/0	2/2	0/0	1/0	2/0	0/0	0/0	0/0	96.55	97.83	3.0
	Positive control[d]		4/5	6/6	0/0	5/2	8/4	2/2	0/0	0/0	73.14	90.00	14.0**
-	Reference control[c]	6 (treatment)	0/0	0/0	2/2	0/0	2/1	0/0	0/0	0/0	100.00	97.33	3.5
	Solvent control	18 (recovery)	0/1	2/0	0/0	0/1	1/1	0/1	0/0	0/0	100.00	96.00	2.5
	64		0/2	0/4	0/0	1/1	2/0	0/0	2/2	0/0	81.90	96.00	3.0
	32		0/0	2/1	2/0	0/1	0/2	0/0	0/2	0/0	86.83	91.00	3.5
	16		0/1	2/0	0/0	0/0	1/0	0/1	0/0	0/0	92.26	95.00	2.0
	Positive control[e]		5/7	12/11	10/17	6/3	11/13	3/2	2/0	2/3	90.32	88.00	39.5**
-	Reference control[c]	22 (treatment)	0/0	0/0	2/0	0/0	1/2	0/0	0/0	0/0	100	94.50	2.0
	Solvent control	2 (recovery)	0/1	2/0	0/0	1/0	2/1	2/0	0/0	0/0	99.52	96.00	3.5
	64		0/0	2/0	2/1	1/0	2/2	0/0	0/1	0/0	76.18	97.00	4.0
	32		0/1	0/0	0/0	0/1	2/2	0/3	2/0	0/0	86.11	92.00	3.5
	16		0/0	0/2	0/0	0/0	2/2	0/0	0/0	0/0	94.59	93.00	3.0
	Positive control[e]		8/7	14/11	23/19	12/7	14/17	3/5	0/2	0/4	89.44	88.00	53.0**

+, With S9 metabolic activation; -, without S9 metabolic activation.

CTG, chromatid-type gap; CTB, chromatid-type break; CTE, chromatid-type exchange.

CSG, chromosome-type gap; CSB, chromosome-type break; CSE, chromosome-type exchange.

PP, polyploidy; ER, endoreduplication.

RPD = No. of population doublings in treated cultures/No. of population doublings in control cultures×100 (%); population doubling = [log (Post-treatment cell number ÷ Initial cell number)] ÷ log2.

MI = cell counts of metaphases/cell counts of flask×100 (%), duplicate cultures examined.

** Significantly different from the control at $p < 0.01$ (based on Fisher's exact test).

[a] Cell number in flask 1/cell number in flask 2, 100 metaphases examined per culture, duplicate cultures used.

[b] Gaps excluded.

[c] The reference control item was complete culture medium.

[d] The positive control was CPA (28 μg/ml).

[e] The positive control was EMS (6 μl/ml).

Supplementary information for methods

Synthesis and identification of CE. Emodin (4.46 g, 14.8 mmol) was suspended in a solvent consisting of acetic acid (300 ml) and concentrated hydrochloric acid (30 ml) and was stirred at 85°C for approximately 3 min. Then, 30% H_2O_2 (8×0.2 ml, 16 mmol) was added 8 times every 5 min. 20 min after the final addition, the starting material was completely converted (TLC monitoring with CH_2Cl_2: MeOH: acetic acid = 9:1:0.025). The mixture was cooled to room temperature and then poured into water (250 ml). The resulting yellow precipitate was collected by suction filtration and was washed twice with cold water (50 ml). After drying in a vacuum oven, CE was obtained as a yellow solid (2.7 g, 60%). HPLC was used to confirm the purity of CE to be ≥95%.

Fluorescence titration experiment. *Micrococcus luteus* genomic DNA was diluted in 10 mM Tris-HCl buffer (pH 7.2). PI and DAPI were diluted in PBS (pH 7.2) to final concentrations of 1 mg/ml and 5 mg/ml, respectively, and then stored in the dark at 4°C before use. CE was diluted in DMSO to a concentration of 0.2 mM before use. As a solvent control, 20 μl of DMSO was added to a sample. Graduated concentrations of PI or DAPI were added into the DNA Tris-HCl dilutions until the fluorescence identities of the system no longer increased. Next, increasing amounts of CE and emodin were added to the DNA Tris-HCl dilutions. All the samples were scanned with a Hitachi FL spectrophotometer F-7000. The quenching constant (K_{SV}) of CE was estimated by the Stern-Volmer equation (S1):

$$\frac{F_0}{F} = 1 + K_{SV}[\mathrm{Q}] \qquad \text{(S1)}$$

where F_0 and F are the fluorescence intensities of the PI-DNA complexes in the absence and presence of CE, respectively; K_{SV} is the Stern-Volmer dynamic quenching constant; and [Q] is the concentration of CE. The Stern-Volmer equation was used to determine the K_{SV} by linear regression of a plot of $\frac{F_0}{F}$ against [Q].

The apparent binding constant (K_a) and the binding stoichiometry (n) were also estimated by equation (S2):

$$\mathrm{Log}\,\frac{F_0 - F}{F} = \mathrm{Log}K_a + \mathrm{nLog}[\mathrm{Q}] \qquad \text{(S2)}$$

Based on the binding constants at 293 and 308 K, the enthalpy change ΔH, the entropy change ΔS and the free energy change ΔG for a binding reaction were calculated by the Vant't Hoff equation (S3) and Gibbs-Helmholtz equation (S4):

$$\mathrm{Ln}\left(\frac{K_{a2}}{K_{a1}}\right) = \left(\frac{1}{T_1} - \frac{1}{T_2}\right)\frac{\Delta H}{R} \tag{S3}$$

$$\Delta G = \Delta H - T\Delta S = -RT\ln K_a \tag{S4}$$

where R is gas constant and T is temperature (Kelvin).

The agarose gel electrophoresis of DNA treated with CE. The Salmon sperm DNA was diluted in Tris-HCl buffer (10 mM, pH 7.2) at a concentration of 20 μg/ml. CE was dissolved in DMSO at a concentration of 64 mg/ml and stored at -20°C. 20 μl solution containing 100 ng DNA and graduate concentrations of CE were incubated at 37°C for 30 min. Samples were resolved by electrophoresis on 0.8% agarose gel for electrophoresis. After electrophoresis, the gels were stained by 0.5 mg/ml ethidium bromide, and visualized with UV light.

***In vitro* cytotoxicity assay of CE.** Chinese hamster lung (CHL) fibroblast cells (inoculated concentration of approx. 5×10^4 cells/ml) were cultured in 96-well plates and incubated at 37 with 5% CO_2 overnight and then were treated with CE or emodin at different concentrations for 24 h. The 3-(4,5-dimethylthiazol-2-yl)-2,5-diphenyltetrazolium bromide (MTT) assay were performed to evaluated the cell viability. Absorbance of each well was measured at 570 nm in a microplate reader (Versa 800). Untreated cells (cells plus medium) were tested as a negative control, which represented 100% viability. Experiments were performed in triplicate.

***In vitro* chromosomal aberration assay of CE.** The assay was performed in accordance with OECD Testing Guideline 473 (OECD, 2014). The Chinese hamster lung (CHL) fibroblast cell line used in the experiment was CHL/IU (ATCC CRL-1935) from Sichuan center for disease control and prevention, P.R. China. The cells were subcultured and maintained to use in the study. Cells were stored in liquid nitrogen and cultured for at least 7 days after thawing. Cells were assessed for proliferation rate (15-h doubling time) and karyotype prior to the initiation of the study. CPA and EMS were used as positive control respectively for the test with and without exogenous metabolic activation. CE was dissolved and temporarily stored in DMSO at a concentration of 12.8 mg/ml. The vehicle used for each sample was the complete culture medium. The complete culture medium added with 0.5% (vol：vol) DMSO was used as solvent control.

The metabolic activation system consisted of S9 fraction and cofactor-I ($MgCl\cdot6H_2O$, KCl, glucose-6-phosphate, NADPH, NADH, and sodium phosphate buffer). S9 fraction was prepared from the livers of male Sprague-Dawley rats pretreated with Aroclor 1254. The S9 mix containing 50 μl of S9 in 1 ml solution was prepared according to standard methods. After addition of the S9 mix solution to the culture medium, the final

concentration of S9 was 5% (0.15 ml S9 in 3 ml culture medium).

In both the range-finding and main studies, an appropriate volume (depending upon the addition of S9-mix) of CE and the positive controls were added to the cell culture and incubated for either 6 h (both in the presence and absence of S9 mix) or 22 h (in the absence of S9 mix only). The RPD, an index of inhibition of cell growth, was calculated to estimate the cytotoxicity of CE with equations S1 and S2:

$$RPD = \left(\frac{\text{Number of Population doublings in treated cultures}}{\text{number of Population doublings in control cultures}}\right) \times 100(\%) \qquad \text{(S1)}$$

$$\text{Population Doubling} = \log\left(\frac{\text{Post - treatment cell number}}{\text{Initial cell number}}\right) \div \log2 \qquad \text{(S2)}$$

In main studies, the CHL cells were seeded into a 25 ml flask at concentration of 1.2×10^4 cells/ml and cultured at 37°C with 5% CO_2 for 3 days. After treatment with test compound for 6 or 22 h, the culture medium was aspirated and the cells were washed with 5 ml calcium and magnesium free phosphate buffer. 5 ml of fresh culture medium was then added and cultured until the collection of mitotic cells. Approximately 22 h after the start of the incubation, 50 μl of colchicine solution was added to each culture. The cultures were then incubated for a further 2 h. Mitotic cells were collected by gentle shaking. Cells were centrifuged and resuspended in 0.075 M KCl for 10 min at room temperature. The cells were fixed by addition of 5 ml of a solution of methanol and acetic acid (3:1). A drop of the suspension was placed on a glass slide. Slides were stained by 5% Giemsa buffered solution, and then rinsed and dried. Two slides for each flask were prepared. The remaining cell monolayer in each flask was trypsinized and counted to calculate RPD.

Using a microscope, 100 well-spread chromosomes in metaphase were selected for each specimen, with a total of 200 cells/concentration (two slides) evaluated. The frequencies of aberrant metaphases were subject to statistical analysis. The statistical tests included comparison of the negative control group with the treatment group (χ^2 test and Fisher's exact test) and comparison of the negative controls with the positive controls (Fisher's exact test).
